# Characterising Ecosystem Composition, Structure and Function of Alternative Stable States in Temperate Forests of South‐Eastern Australia

**DOI:** 10.1111/gcb.70895

**Published:** 2026-05-05

**Authors:** Aaron E. Heap, Tom A. Fairman, Trent D. Penman, Lauren T. Bennett

**Affiliations:** ^1^ FLARE Wildfire Research Group, School of Agriculture, Food & Ecosystem Sciences The University of Melbourne Melbourne Victoria Australia

**Keywords:** alternative states, ecosystem function, ecosystem transformation, *Eucalyptus*, fire regimes, forest structure, short‐interval wildfire

## Abstract

Increasingly frequent and intense wildfires threaten to push ecosystems beyond their ecological thresholds. Fire‐adapted forests can be vulnerable to ecosystem conversion under shortening fire‐return intervals. To evaluate the impacts of fire regime change, we contrasted *Eucalyptus* forests that retained canopy cover under tolerable regimes (reference forests) with alternative states of markedly reduced canopy. These alternative states established following short‐interval wildfires and have persisted through subsequent fires. Alternative states were dominated by more uniform distributions of small trees (< 20 cm diameter) than reference states, with 97% fewer large trees (> 20 cm diameter). These changes in structure translated into declines in several key ecosystem functions. The loss of large, hollow‐forming trees and limited *Eucalyptus* regeneration indicate long‐term constraints on habitat availability for hollow‐dependent fauna. Coarse woody debris mass was 38% lower in alternative states, indicating decreased resources for detritivores, fungi, and ground‐dwelling mammals. Aboveground carbon stocks were reduced by 59%, with a much higher proportion (98%) stored in small trees than reference states (20%). Lower fire tolerance of small trees suggests a greater vulnerability of carbon stocks to subsequent fires in alternative states compared to reference states. These findings highlight the long‐term ecological consequences of altered fire regimes. In addition, emphasising the management and restoration of forest structural integrity is essential to maintain ecosystem functions in fire‐prone landscapes that are increasingly vulnerable under changing climates.

## Graphical abstract

Alternative stable states established following repeated short‐interval fires in resprouting *Eucalyptus* temperate forests were associated with declines across multiple ecosystem functions. For example, carbon storage potential was significantly reduced (b: *p* < 0.001), with alternative states storing substantially less carbon than reference forests, particularly in the live (a: *p* < 0.001) and dead (*p* < 0.05) carbon pools. Moreover, the shift to a structure dominated by small stems (< 20 cm DBH; d: *p* < 0.001) makes live carbon stocks more vulnerable to subsequent fires, increasing the likelihood that these systems function as carbon sources rather than carbon sinks.

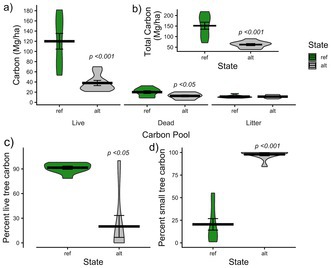

## 1 Introduction

Changing fire regimes across the world are driving ecosystem state changes (Cesca et al. 2014; Kelly et al. 2023; Peinetti et al. 2024; Suding et al. 2004). Fire regimes refer to the extent, intensity, and the frequency of wildfires within a region or ecosystem type (Bradstock 2010; Gill 1975; Krebs et al. 2010). In recent decades, fire regimes in Canada, the western United States, and Australia have departed from historical norms, with documented increases in area burned, fire frequency and intensity, and longer fire seasons (Bowman et al. 2020; Canadell et al. 2021; Higuera and Abatzoglou 2021; Jones et al. 2022; Kasischke and Turetsky 2006; Linley et al. 2022; Turco et al. 2019; Whitman et al. 2022). These shifts are associated with changing climate conditions and altered fire weather. For example, increasingly warmer and dryer climate conditions occurring in British Columbia, Canada since 1986 coincide with increasing magnitude of large fires and annual area burned (Collins et al. 2025). Moreover, Australia's 2019/2020 fire season was unprecedented in the extent burned, radiative power, and coincided with more dangerous fire weather and record lows of fuel dryness (Abram et al. 2021; Boer et al. 2020; Collins et al. 2022; Wang et al. 2022).

Changes in fire regimes can result in conversion of forests to an alternative state (Falk et al. 2022; Scheffer and Carpenter 2003). Many species are reliant on wildfire to complete their life cycles. Fire‐adapted traits support post‐fire recovery towards an ecosystem that resembles the pre‐fire state (Keeley et al. 2011; Rundel et al. 2018). However, regeneration can fail if fire regimes exceed an ecological tolerance, leading to the establishment of a community that no longer resembles the pre‐fire ecosystem in plant composition or structure (Falk et al. 2022). Conversely, in some ecosystems, it has been suggested that fire frequencies exceeding historical norms have had positive effects by limiting invasive woody vegetation and preventing its encroachment from forest margins into meadows (Norman and Taylor 2005). The continued persistence of these communities leads to their recognition as an alternative stable state (Coop et al. 2020; Enright et al. 2015; Falk et al. 2022; Scheffer and Carpenter 2003; Seidl and Turner 2022). For example, shorter fire‐return intervals than the dominant tree species' juvenile phase have converted conifer forests to ecosystems dominated by deciduous species and state conversions to shrublands or grasslands (Dwomoh and Wimberly 2017; Buma et al. 2022; Walker et al. 2025). In forests dominated by resprouting *Eucalyptus*, short‐interval wildfires have increased tree mortality, reduced seedling recruitment, and led to structural and compositional changes in resprouting sclerophyll forests (Fairman et al. 2017, 2019).

Understanding the impacts of altered fire regimes on forest ecosystems requires a clear assessment of the magnitude of change associated with conversion to alternative states. Alternative ecosystems are often considered degraded in terms of ecosystem functions and services (Adams 2013; Enright et al. 2015; Lavorel et al. 2015; Lindenmayer et al. 2021). Short‐interval fires can simplify structure (Heap et al. 2025), reduce niche availability and exclude species dependent on complex forest structures for refugia (MacArthur and MacArthur 1961; Sukma et al. 2019). Conversion to alternative states is often characterised using changes to ecosystem composition, structure and function (Bielski et al. 2021; Coop et al. 2020; Knox and Clarke 2012; Tiribelli et al. 2018) and consequential changes to productivity, carbon storage, transpiration rates and increased surface water (Coop et al. 2020; Lakmali et al. 2022; Zinnert et al. 2021). These same attributes—composition, structure and function—are also commonly used in assessments of ecosystem quality (Gann et al. 2019; Hansen et al. 2021; Parrish et al. 2003; Tierney et al. 2009).

Characterisation of forest attributes provides a framework for assessing degradation by calculating the degree to which an ecosystem has diverged from the non‐converted ‘reference ecosystem’ (Gann et al. 2019). Furthermore, by evaluating multiple ecosystem functions, managers can better understand how alternative states influence broader landscape ecosystem processes and resilience (Aronson et al. 2017; Stanturf et al. 2014). These data can, for example, help managers identify thresholds to passive recovery such as limitations arising from the loss of propagule sources or the distance of alternative states from propagule sources (Booth 2017). They can thus be used to guide required levels of intervention and to provide empirical evidence to set goals and establish targets for restoring alternative states towards the preferred reference ecosystem.

An increase in the frequency and extent of high‐intensity fires increases the risk of Australia's fire‐tolerant resprouter forests being converted to alternative states (Kelly et al. 2023; Nolan et al. 2021). Ecosystems altered by inappropriate fire regimes remain under‐described, with evaluations often limited to changes in a single function or prominent societal values (Stanturf et al. 2014). Current understanding of the differences in ecosystem functions and services of alternative states versus reference forest is largely assumed and semi‐quantitative (Colloff et al. 2016; Lavorel et al. 2015). To date, much of the research on alternative states in Australia has focused on state changes between grasslands, savannah, and tropical forests (Cheng et al. 2025; Mata et al. 2025). Very few studies have examined the composition and structure of alternative states associated with *Eucalyptus* forests in southeastern Australia (Ashton and Chappill 1989; Brown et al. 2021; Burton et al. 2019; Heap et al. 2025). Specifically, the implications of state change to ecosystem function remain under examined in fire‐tolerant resprouter forests. Quantifying any associated ecosystem degradation is essential for guiding management and restoration (Enright et al. 2015; Gann et al. 2019; Kelly et al. 2023; Nolan et al. 2021). The aim of this study was to quantitatively assess the extent of changes in species composition, structural diversity, and ecosystem functions resulting from short‐interval fire‐induced shifts in stable states. This study quantifies how alternative states resulting from repeated short‐interval fires in temperate, mixed‐species resprouting eucalypt forests differ from reference states with respect to species composition, structural diversity, and key ecosystem functions, including carbon storage and habitat resource availability.

## 2 Methods

### 2.1 Study Area

**FIGURE 1:**
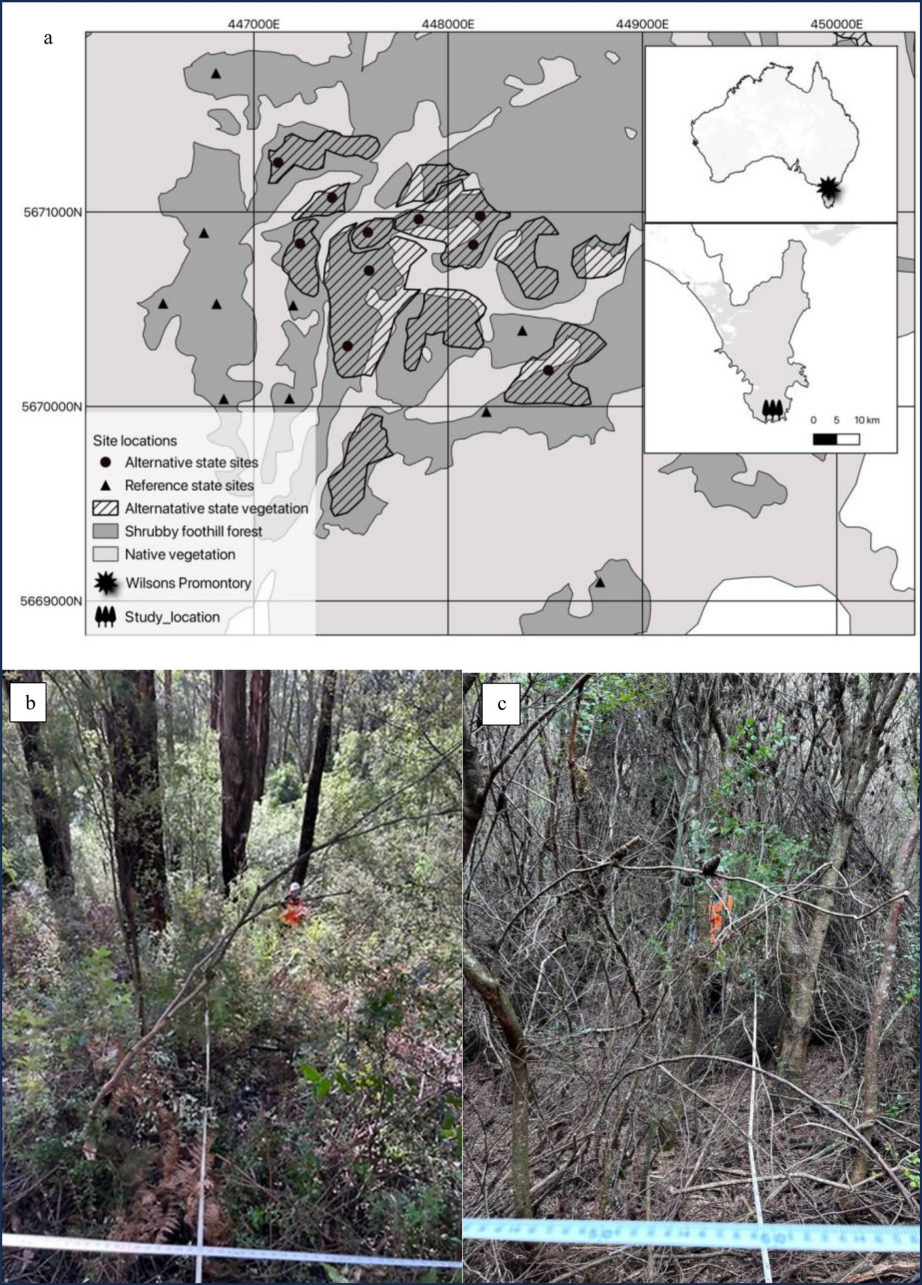
(a) Location of plots within Wilsons Promontory National Park, Victoria, Australia, including the extent of native forest and of Shrubby Foothill Forest. Areas of forest in an alternative state are mapped in crosshatch and were first identified by Davies and Oates (1999) and refined by Heap et al. (2025) using 2024 satellite imagery (Google 2023). Symbols distinguish sites classified as alternative states and those classified as reference states. (b) Photo of transect in a reference forest. (c) Photo of the transect set up in alternative state.

Field surveys were undertaken in temperate mixed‐species eucalypt forests of Wilsons Promontory National Park, located ~200 km from the city of Melbourne, Victoria in south‐eastern Australia (Figure 1a). The Promontory is strongly influenced by a maritime climate due to its narrow geographic profile. Mean monthly temperatures range from a minimum of 8.4°C to a maximum of 20.6°C (1877–2025) and mean annual rainfall is approximately 1057 mm (1872–2025) (Australian Government, Bureau of Meteorology 2024). The variable topography and geology of the Park support an extensive variety of ecosystems in a relatively small spatial extent (~480 km^2^; DEECA 2025).

Eucalypt‐dominated vegetation covers approximately 46% (230 km^2^) of the Park (DCCEEW 2023). This study focuses on the Ecological Vegetation Class (EVC)—Shrubby Foothill Forest (SFF; Figure 1b) (DEECA 2025). Shrubby Foothill Forest is widespread throughout Victoria; within Wilsons Promontory, this forest type occurs across approximately 3433 ha, at lower elevations (9 to 659 m asl.), and on northerly and westerly slopes, which tend to be the driest in the local environment (DEECA 2022; Heap et al. 2025). According to the EVC ‘benchmark’ for SFF specific to the Wilsons Promontory bioregion, this vegetation type is characterised by a canopy of fire‐tolerant, resprouting *Eucalyptus obliqua* and *E. baxteri* , up to 25 m tall, with a density of ~20 large trees/ha (> 70 cm diameter at breast height [DBH; measured at 1.3 m]). The understorey is comprised of narrow‐leaved shrubs with occasional ferns, graminoids, and herbs (DEECA 2025).

#### 2.1.1 Fire History

The study landscape has been occupied for at least 6500 years by Indigenous Australians who regularly applied fire to the landscape (Collett 1994; Coutts 1970; Ellender 2002). European records of fire history for the region begin in 1863 and several fires were associated with European settlement and land clearance (Chesterfield 1998). Resprouter‐dominated eucalypt forests across south‐eastern Australia are typically characterised by moderate‐intensity, spring–summer wildfires, with fire return intervals of 5 to 20 years (Murphy et al. 2013). However, historical fire records of fires within Wilsons Promontory described in Heap et al. (2025) and Ashton and Chappill (1989) suggest substantial spatial and temporal variability. For example, multiple extensive fires throughout the 20th century led to ~3000 ha of *Eucalyptus* forest to be burned at intervals shorter than the documented recovery time required for *Eucalyptus* species to re‐establish and reach reproductive maturity (Spencer et al. 2020). The overlapping wildfires in 1939 and 1951 are thought to have caused the widespread loss of the eucalypt overstorey and the conversion of approximately 1051 ha of forest to a non‐forest alternative state (Ashton and Chappill 1989; Chesterfield 1998; Davies and Oates 1999; Heap et al. 2025). These alternative states have persisted, with *Eucalyptus* remaining absent, despite more than 25 years without large wildfires, and following extensive wildfire events in 2005 and 2009, each burning approximately half of the park (Heap et al. 2025). This highlights the limitations of *Eucalyptus* propagules repopulating when the seedbed is cleared of competition and that these alternative states are in fact a stable state (Coop et al. 2020; Petraitis and Latham 1999). Although this study focuses on alternative states of SFF (~352 ha), these states also occur through areas of forests classed as Wet (~664 ha) and Damp forests (~323 ha) (Davies and Oates 1999; Heap et al. 2025). Fairman et al. (2016), Enright et al. (2015), and Keeley et al. (1999) described the mechanisms of how short‐interval fires can lead to declines in resprouting capability and seedling regeneration in Australian forests; mechanisms that are also applicable to the loss of the eucalypt overstorey at Wilsons Promontory.

#### 2.1.2 Alternative States

Visible fallen stems in aerial photography of Wilsons Promontory from 1941 confirm that the patches of alternative states within the expected SFF distribution once supported large canopy trees, prior to the widespread fire of 1951. The conversion of forests to alternative states are of concern for land managers and the extent of the alternative areas was first mapped in 1999 using fire history and ariel photography (Davies and Oates 1999). These maps were later refined using 2024 Google satellite imagery (Heap et al. 2025) and were used to identify the extent of alternative states in the landscape for this study. In the absence of the eucalypt overstorey, Ashton and Chappill (1989) describe the change of dominant species in alternative states of Wilsons Promontory. Recent analysis by Heap et al. (2025) based on LiDAR data collected in 2008–2009, indicated that areas classified as alternative states (Figure 1c) were characterised by significantly reduced vegetation height and overstorey cover compared to surrounding ‘reference’ vegetation (Figure 1b; i.e., same EVC, not disturbed by short‐interval fires and considered closer to the benchmark description).

### 2.2 Site Selection

A total of 20 survey sites were evenly distributed across mapped locations of alternative and reference forest states within the National Park SFF distribution using Geographic Information System (GIS) software. To limit edge effects and ecotones, sites were selected to be minimum of 50 m from roadsides, walking tracks, and forest ecotones by analysing satellite imagery and GIS maps. To prevent spatial confounding, sites were at least 150 m apart. Once in the field, sites were assessed to ensure fit with the SFF distribution (i.e., north or west‐facing slope, at least 50 m from a gully line or waterway). At each site, aspect, elevation and slope at the plot centre and the identity and estimated height of the *Eucalyptus* overstorey species were recorded.

### 2.3 Field Measures

Site assessments were undertaken between February 2023 and March 2024. At each site, two 50 m transects (oriented north–south, NS, and east–west, EW) were positioned in a crosshair formation at the pre‐selected plot centre. Nested plots were positioned along the transects, with radii of 10 and 25 m from the plot centre (Supporting Information S1). Along both the NS and EW transects, twelve 1 m^2^ subplots were systematically positioned at 3 m intervals between 8 and 41 m. At the centre of each 1 m^2^ subplot, vegetation structure was assessed using a 2‐m vertical touch‐pole. In addition, overstorey cover was assessed as cover above 2 m high using the *Canopy% Cover* application on an iPhone 14 (Public Interest Enterprises 2021). Vegetation presence or absence was recorded at 10‐cm intervals up the pole, based on contact with any part of the plant (Pickering et al. 2023). Within each subplot, vascular plant species richness was recorded, and the percentage cover of grasses, herbs, small shrubs (< 1.3 m), medium shrubs (> 1.3 m), and ferns were visually estimated to the nearest 5%. Estimates were recorded separately for live and dead vegetation. All assessments were made by the same observer, with frequent discussion and checks with team members to ensure consistency of the visual estimates within and among sites. Shrubs were defined as multi‐stem lifeforms with a maximum stem diameter of 2.5 cm. In addition, percent cover of litter within the 1 m^2^ sub‐plots was recorded to the nearest 5% and the litter depth measured using the method outlined in the Overall Fuel Hazard Guide (Hines et al. 2010). Any species observed within the larger 25‐m radius plot but not recorded in the 1‐m^2^ subplots were also noted and included in species richness estimates. The presence and identity of exotic plant species within the plot were also recorded.

The numbers of live and dead tree stems were recorded across four size classes: seedlings (< 2.5 cm diameter), small stems (2.5–10 cm DBH), medium stems (10–20 cm DBH), and large stems (> 20 cm DBH). Seedlings were counted by species within the 1 m^2^ sub‐plots. Small and medium stems were counted within the northeast (NE) and southwest (SW) quadrants of the circular 10‐m radius subplot, and the DBH of all large trees was measured by species within the 25‐m radius plot. Following the methodology of Fairman et al. (2019), any stems resprouting below breast height were measured individually, and the status of each stem (live or dead) was recorded. Dead trees with a trunk height ≥ 1.3 m were classed as dead trees or stags. Stumps were classed as dead trees < 1.3 m height. The height and cross‐sectional diameter of stumps were measured at a height of 30 cm, with an estimate made of the proportional cross‐sectional area remaining.

Coarse woody debris (CWD; fallen wood no longer rooted in the soil and > 2.5 cm in diameter) was measured along each 50‐m transect using the line‐intercept method outlined in Van Wagner (1968). The cross‐sectional diameter of each CWD piece intersecting the transect line was measured, and the remaining cross‐sectional area was estimated to the nearest 5%. Each piece of debris was classified into one of five decay classes, following the criteria established by Aponte et al. (2014). These ranged from D1 no decay to D5 substantially decayed and later grouped as sound (D1–D3) and rotten (D4–D5).

Four litter samples were collected at fixed locations 10 m from the plot centre along each transect, using a metal quadrat with an area of 0.09 m^2^. Litter was defined as dead or decomposing plant material < 2.5 cm in minimum cross‐sectional diameter, above the mineral soil and included leaves, twigs, small branches, and bark (IPCC 2003). Coarse woody debris, rocks, and living plant material were removed before drying the samples in an oven at 40°C to a constant mass. The dried samples were then sieved to remove soil and the remaining sample weighed.

### 2.4 Ecosystem Attributes

To assess variation in ecosystem quality we compared elements of ecosystem composition, structure and function between the reference and alternative forest states. The attributes here were chosen to encompass as many ecosystem functions as possible within the constraints of the study's timeline and resourcing. The functions chosen were informed by the goals of the State Park Service to understand the differences in carbon storage and the availability of habitat resources for wildlife. A suite of metrics was calculated using established methods and supported by published literature. All calculations were conducted in the R processing environment (version 4.4.2; 2024‐10‐31; R Core Team 2024). A detailed summary of each attribute, including variables calculated, methods used, and supporting citations, is provided in Table 1.

**TABLE 1:** Detailed summary of the ecosystem attributes assessed, and the sub‐attributes compared between states. The table includes the calculated metrics, a description of the calculation method and associated citation.

Ecosystem attribute	Sub‐attribute	Metric	Calculation method	References
Species composition	Species richness	Number of species	Count of unique species per 25‐m radius plot	
	Understorey species composition	Bray–Curtis dissimilarity calculated from proportional abundances of genus	Community composition of live species assessed using non‐metric multidimensional scaling (NMDS) based on Bray‐Curtis dissimilarities of proportional abundances (metaMDS, *vegan*; *k* = 2 dimensions, 100 random starts). Group differences assessed with PERMANOVA (adonis2, 999 permutations)	(Anderson 2005; Oksanen et al. 2025; Venables and Ripley 2002)
	Indicator species of state	Mean proportional abundance of each genus	Proportional abundances of genus compare between states, retaining taxa present in ≥ 20% of sites. Indicator species analysis (IndVal.g; 999 permutations, BH correction) and SIMPER (Bray‐Curtis dissimilarities; 999 permutations) used to assess fidelity and quantify genus' contribution to community separation	(Clarke 1993)
	Floristic composition relative to benchmark	Species presence/absence	Comparison of species presence or absence with the species list in the Ecological Vegetation Class benchmark for the study area bioregion	(DEECA 2025)
Structural diversity	Growth form richness	Number of growth forms	Count of unique plant growth forms per site using classifications from AusTraits and VicFlora	(Falster et al. 2021; VicFlora 2025)
	Composition of understorey growth forms	NMDS ordination (Bray‐Curtis)	NMDS based on proportion of understorey growth forms from cover/count data PERMANOVA used to test for significant differences as described in understorey species composition	(Anderson 2005; Oksanen et al. 2025; Venables and Ripley 2002)
	Indicator growth forms of state	Mean proportional abundance of each growth form	As per indicator species of state above	(Clarke 1993)
	Species richness by growth form	Number of species within growth form classification	Count of unique species per growth form, per site	
	Growth form frequency	Proportion of plots with growth form present	Proportion of plots within each site where a given growth form was present; classifications from AusTraits and VicFlora	(Falster et al. 2021; VicFlora 2025)
	Structural diversity of stems	Basal area; Stem Density; Weighted Mean Diameter at Breast Height (DBH); Weighted Coefficient of Variation (COV) of DBH	Metrics calculated for total, live, and dead stems across all DBH classes; weighted means used to correct for variation in sampling effort. Weighted mean diameter calculated by multiplying each size class midpoint by number of individuals per hectare in that class, summing across classes, and dividing by the total number of individuals per hectare	(Fairman et al. 2019; Lindroos et al. 2024)
	Structural diversity large stems (> 20 cm DBH)	Stem Density; Weighted Mean DBH; Weighted (COV) of DBH	As structural diversity of stems above but restricted to stems > 20 cm DBH only. COV calculated separately for live and dead stems	
	Structural complexity index	Composite score	Sum of standardized values for overstorey cover (> 2 m), litter depth, and vegetation cover of 0–20 cm, 20–50 cm, 50–100 cm, and 100–200 cm strata	(Sukma et al. 2019; Swan et al. 2015)
	Vegetation strata dissimilarity	Gower's index, PCA axis scores	Gower's dissimilarity index calculated for each stratum listed in the structural complexity index. A value of 0 indicates homogeneity of the strata. Strata variables were standardized (mean = 0, SD = 1) and first principal component (PC1) from analysis was used as a single composite index of strata consistency for each site	(Gower 1971; Sukma et al. 2019)
	Coarse woody debris	Size class counts; mean mass, diversity of piece size; % of sound/rotten piece by mass	Mass estimated per piece based on decay class‐adjusted volume; size class diversity assessed by COV of piece mass; proportion of sound mass relative to total mass and proportion rotten mass relative to total mass	(Fairman et al. 2017; Grierson et al. 1992; Van Wagner 1968)
Ecosystem function	Carbon storage	Total carbon (Mg/ha); carbon in live biomass, deadwood and litter pools	Biomass *estimated from allometric equations* (Table S2) *and converted using* 0.50 carbon factor. Processed litter samples were weighed and extrapolated to Mg/ha. For dead trees a 0.75 multiplier was used for foliage and branch loss and 0.80 multiplier for wood decay. Coarse woody debris carbon was estimated using a volume equation converted to mass using an average density of either sound or rotten dead eucalypt wood	(Aponte et al. 2020; Bennett et al. 2017; Fairman et al. 2022; Feller 1980; Grierson et al. 1992; IPCC 2021; Van Wagner 1968)
	Carbon stability	Percent live tree carbon (%), percent small tree carbon (%)	Proportion of live tree carbon relative to the total carbon contained in both live and standing dead trees, and proportion of carbon stored in small trees < (20 cm DBH) relative to the total live tree carbon	(Bennett et al. 2017; Fairman et al. 2022)
	Hollow availability	Trees/ha with *p*(hollow) > 0.5	Based on species‐specific hollow–DBH models established using state forest resource inventory data from forests closest to the study area and relevant species to the study (Supporting Information S3)	
	Floral and seed resource richness	Species count by biotic and abiotic seed dispersal/pollination modes	Species assigned seed dispersal/pollination modes via AusTraits. The number of species dispersed or pollinated by invertebrate, vertebrate, were then calculated for each site	(García and Martínez 2012; Kitahara et al. 2008; Ollerton 2017)

#### 2.4.1 Species Composition

Species composition was evaluated through species richness, ordination techniques, and comparisons with EVC benchmarks (Table 1). Understorey community composition (excluding *Eucalyptus* spp.) was compared between states using the MASS package to perform Non‐metric Multidimensional Scaling (NMDS) based on Bray‐Curtis dissimilarities (Venables and Ripley 2002). Species abundance was expressed as site‐based proportions derived from percentage cover and count data standardised using the VEGAN package. Significant differences between states were tested using Permutational Multivariate Analysis of Variance (PERMANOVA) (Anderson 2005). Key genera responsible for the separation in ordinal space were identified by comparing mean proportional abundances between states and applying a prevalence filter (≥ 20% of sites). Similar percentage (SIMPER) breakdown analysis was conducted to identify species indicative of either an alternative or reference state and quantified for their contributions to overall community separation (Clarke 1993).

#### 2.4.2 Structural Diversity

Structural diversity was assessed by examining growth form composition, variability in structural metrics of standing and dead wood, vegetation complexity, and consistency of strata cover across sites (Table 1). The growth forms of recorded species were extracted from the AusTraits database (Falster et al. 2021). Data gaps for growth forms or species absent from the database were filled using the VicFlora herbarium (VicFlora 2025). Descriptive statistics were used to summarise site characteristics. NMDS was used to compare the composition of growth forms between states as described in the species composition above, and to quantify indicative growth forms of each state. In addition, we compared species richness within each growth form and assessed the frequency of growth forms across sites.

Structural diversity measures of standing and deadwood were calculated from DBH measurements of live and dead stems such as mean stems/ha, mean DBH, and the coefficient of variation (COV) of DBH and of large trees (stems > 20 cm DBH) between states (Table 1). The fallen stems (coarse woody debris) were treated in a similar manner using piece counts in 10 cm‐diameter size classes. The diversity of piece sizes was evaluated using the COV of piece mass. For each site, the biomass of rotten (decay class 3–5) and sound pieces (decay class 1 and 2) was compared. Structural complexity, defined here as an additive measure of vegetation cover and litter depth, has been linked to increased mammal occurrences and functional diversity in Australian forests (Sukma et al. 2019; Swan et al. 2015). We used an established methodology to calculate structural complexity which is an additive measure of mean vegetation cover and litter depth (Table 1) (Sukma et al. 2019; Swan et al. 2015). To evaluate consistency in structural composition between states, we used the same strata of the structural complexity index and calculated Gower's dissimilarity matrices for all the strata levels and tested for group separation with Analysis of Similarities (ANOSIM; Clarke 1993) and PERMANOVA, each based on 999 permutations. A Principal Components Analysis (PCA) was performed on scaled structural variables (mean = 0, SD = 1), including vegetation cover across the 0–20 cm, 20–50 cm, 50–100 cm, and 100–200 cm height strata, litter depth, and overstorey cover. PCA loadings were used to interpret the contribution of each stratum to the multivariate gradient, while site scores on the first principal component (PC1) were extracted as an overall ‘PCA index’ to summarise structural composition and compare between states.

#### 2.4.3 Ecosystem Function

##### 2.4.3.1 Carbon Calculations

Above‐ground biomass and carbon stocks were estimated using established methodologies (Table 1; Aponte et al. 2020; Bennett et al. 2017; Fairman et al. 2022). Carbon stocks were calculated for each of the three main aboveground pools: (live) biomass, deadwood (standing and fallen), and litter (IPCC 2003). Biomass of trees of size class 20–50 cm DBH was estimated using species‐specific allometric equations where available; otherwise, generic equations were used for less common species (Supporting Information S2). For trees exceeding 50 cm DBH, the ‘large eucalypt’ equation (Dean et al. 2012) was used, incorporating a decay modifier for internal rot (Roxburgh et al. 2006). Mass of dead trees was adjusted for bark, foliage, and branch loss using a 0.75 multiplier (Feller 1980) and for wood decay using a 0.80 multiplier (Grierson et al. 1992). Small (2.5–10 cm DBH) and medium (10–20 cm DBH) live tree biomass was derived from stem counts multiplied by the biomass of a representative stem of each size class, calculated using the Australian understorey tree allometric equation (Dean et al. 2012). Mass of dead small and medium trees was similarly adjusted with decay multipliers. Stump mass was calculated by multiplying the number of stumps by a representative cylindrical volume (10 cm diameter, 80 cm height) and the average density of sound dead wood (0.459 g/cm^3^) with adjustments for remaining volume (L. T. Bennett et al. 2017). Shrubs species were separated into two size classes medium (≥ 1.3 m height) or small/prostate (< 1.3 m height) shrubs. Biomass of shrubs was determined from cover estimates using the Acacia shrubland equation for medium shrubs or the native chenopod equation for small/prostate shrubs (Grierson et al. 1992). Coarse woody debris (CWD) carbon was estimated using the volume equation of Van Wagner (1968), which was converted to mass using an average density of either sound or rotten dead eucalypt wood (Aponte et al. 2020). Mass per pool (biomass, standing dead wood, CWD, litter; Mg/ha) was converted to carbon mass (Mg/ha) using a 0.5 multiplier (Aponte et al. 2014; Bennett et al. 2017; Fairman et al. 2022).

Carbon stability was estimated using two established indicators relevant to fire‐tolerant forests (Bennett et al. 2017; Fairman et al. 2022). The first—percent live tree carbon—was calculated as carbon stocks in live standing trees > 20‐cm DBH as a percentage of total carbon stocks in both live and standing dead trees of the same size class (Table 1). Carbon stability is assumed to increase with percent live tree carbon since the capacity to recover carbon stocks between fires is comparatively greater if proportionally more carbon is stored in growing trees than decomposing large trees (Bennett et al. 2017). The second indicator was percent small tree carbon, calculated as carbon stocks in live small trees (< 20 cm DBH) as a percentage of live tree carbon stocks in all size classes. This indicator represents the vulnerability of tree carbon stocks to subsequent fire—that is, carbon stability decreases with increasing percent small tree carbon since small trees are more likely to be killed by wildfire (Bennett et al. 2017). The 20 cm tree‐size threshold was selected based on post‐fire mortality of fire‐tolerant eucalypt stems by size class in temperate Australia (Fairman et al. 2019).

##### 2.4.3.2 Habitat Provision

###### 2.4.3.2.1 Probability of Hollow Formation

To evaluate the quantity of hollow‐bearing trees, we compared the probability of hollow occurrences between states calculated using hollow–DBH allometric models (Table 1). The models were developed from data collected through basal area (factor = 3) as part of the State Forest Resource Inventory (DNRE 1999), conducted across Victoria's principal forest management areas. In that assessment, all trees (live and dead) with a DBH greater than 20 cm were assessed for the presence of hollows via ground‐based visual inspection. To establish allometrics that match conditions of our Wilsons Promontory study site, we selected data from three nearby forest management areas: Central, Central Gippsland, and Tambo. The analysis focused on two *Eucalyptus* species commonly found in surveyed sites: *E. baxteri* and *E. obliqua* . In addition, a separate hollow–DBH allometric model was developed for standing dead trees, for which species identity was often unknown. Model parameters and associated summary statistics for each species and tree status (i.e., standing dead) are presented in Supporting Information S3a,b. These allometric relationships were used to estimate the number of trees per hectare with a probability > 0.5 of containing a hollow.

###### 2.4.3.2.2 Floral and Seed Resource Availability

We compared the species richness of plants that rely on biotic pollination and seed dispersal, as a basic comparison of resource availability for pollinators and granivores between states (Table 1). Greater plant diversity has been linked to greater diversity of nectivorous and granivorous species in several studies (García and Martínez 2012; Kitahara et al. 2008; Ollerton 2017). Therefore, species richness of plants requiring insect pollination was used as a proxy to compare resource abundance for pollinators and the concept extended to seed resources for granivorous species (Liss et al. 2013; Potts et al. 2004; Sponsler et al. 2023). Seed dispersal and pollinator groups were extracted from the AusTraits database (Falster et al. 2021) for each plant species recorded in the site surveys. The groups were re‐classified into three categories based on modes of pollination or seed dispersal: invertebrates (insects and ants) vertebrates (vertebrates and birds) and abiotic (wind and passive—i.e. potential for but not requiring a biotic vector).

### 2.5 Data Analysis

We tested the influence of forest state (reference vs. alternative: *n* = 10) on sub‐attribute metrics as response variables. Response variables that met the assumptions of normality using the Shapiro–Wilk test were tested for a statistically significant influence of forest state on the variables using a one‐way ANOVA. Variables that did not meet the ANOVA model assumptions were transformed using either log, arcsine, squared, square root, or boxcox transformations, depending on the form of their distribution. Variables that did not meet the assumptions of ANOVA after transformations were tested using the non‐parametric Kruskal–Wallis test. All statistical tests were run using the R software package (R Core Team 2024).

## 3 Results

This study's field assessments in 2024 are consistent with the lidar‐based descriptions of Heap et al. (2025). Alternative states of SFF had a mean overstorey height of 4.7 m (±0.22 SE) and basal area of 26.4 m^2^/ha (±2.9 SE; Table 2). Overstorey cover (above 2‐m height) of alternative states was 72% (±2.88) compared to 67% (±1.57) in reference states. Field measures of reference states within SFF indicate mean canopy height of 19 m (±1.86), overstorey cover of 67% (±1.57), and mean basal area of 51.3 m^2^/ha (±5.3 *p* < 0.001). An overview of the key sub‐attributes and their directional differences compared to the reference state is summarised in Table 3.

**TABLE 2:** Summary of forest structural metrics for alternative and reference SFF states (mean ± standard error) and the total extent of both within Wilsons Promontory National Park. Mean overstorey heights of the alternative forests are the mean of the 24 × 1 m^2^ subplots along two transects. Mean overstorey heights of reference states were visually estimated in the field.

Metric	Reference states (*n* = 10)	Alternative states (*n* = 10)
Mean overstorey height (m)	19.0 ± 1.86	4.7 ± 0.22
Mean overstorey percentage cover (above 2 m) (%)	67 ± 1.57	72 ± 2.88
Mean basal area (m^2^/ha)	51.3 ± 5.3	26.4 ± 2.9
Total extent within Wilsons Promontory National Park	3433 ha	~352 ha

**TABLE 3:** Summary of the difference in key sub‐attributes between reference and alternative states. Directional arrows indicate if alternative states are statistically significantly higher or lower (⇓) than the reference forest (*p* < 0.05). The double ended arrows (⇔) indicate no statistical difference and * indicates a non‐directional significant difference.

Ecosystem attribute	Sub‐attribute	Difference relative to reference state
Species composition	Species richness	⇔
	Understorey genus composition	*
Structural diversity	Growth form richness	⇓
	Understorey growth form composition	*
	Structural diversity of all stems	⇓
	Structural diversity large stems (> 20 cm DBH)	⇓
	Structural complexity index	⇔
	Vegetation strata consistency	⇔
	Coarse woody debris	⇓
Ecosystem function	Carbon storage	⇓
	Carbon stability	⇓
	Hollow availability	⇓
	Floral and seed resource richness	⇔

### 3.1 Species Composition

Species richness in alternative states was 14.0 (±1.17), while reference states were 15.8 (±1.27 SE), but these differences were not significant (*p* = 0.484). A total of 42 species were recorded to species level across all sites, 34 across the reference states (10 unique) and 31 across the alternative states (6 unique). No exotic species were detected at any of the sites. The main *Eucalyptus* species present were *E. baxteri* , *E. obliqua* , and a possible hybrid of the two. Due to morphological similarities and pending phenological confirmation, hybrids were classified as *E. obliqua* in this study.

**FIGURE 2:**
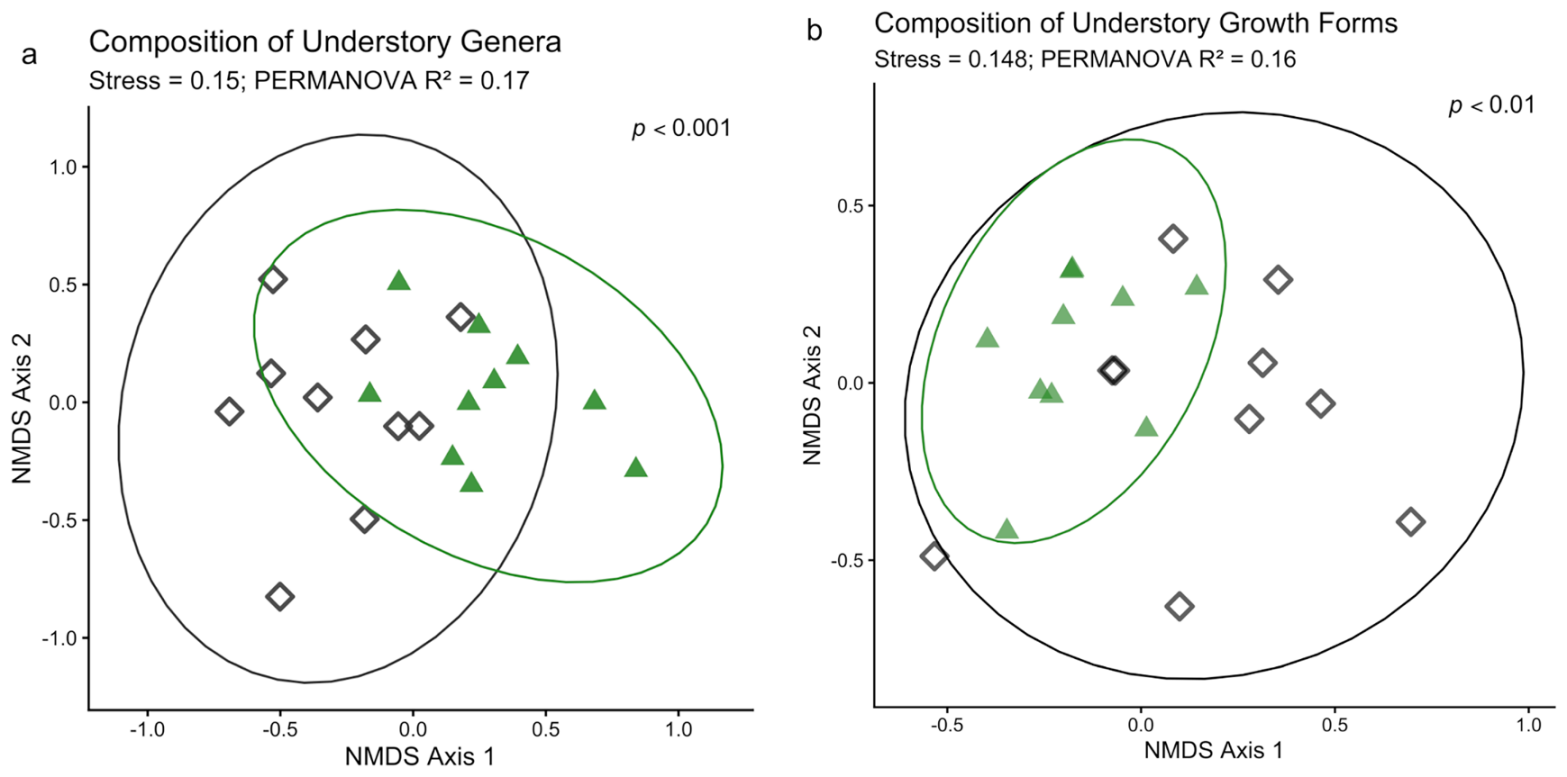
Non‐metric multidimensional scaling (NMDS) ordination of Bray–Curtis dissimilarities among sites of the understorey (small trees, shrubs, ferns, grasses and herbs) composition of genera (a) and growth form (b) between states (Δ = ref, ◊ = alt). Ellipses indicate the 95% confidence intervals. PERMANOVA stress values of 0.15 indicate good representation of the data, although with only ~15% of the variation explained (as indicated by the *R* ^2^ values). *p* values indicate significant differences between reference and alternative states in the composition of genera (a; *p* < 0.001) and growth forms (b; *p* < 0.01).

Multivariate analysis confirmed that understorey community composition differed significantly between reference and alternative states (*R* ^2^ = 0.171, *p* = 0.001; Figure 2a). Differences in state explained 17.1% of the variation in community structure, with most variation occurring within states. Several genera were identified as key drivers of compositional differences between reference and alternative states. Alternative sites were characterised by higher presence of *Billardiera*, *Kunzea*, *Acacia*, *Pomaderris*. *Billardiera* was significantly more abundant in alternative sites (*p* < 0.05) occurring in all sites and 90% of reference sites. *Kunzea* (0.014 ± 0.013 alternative vs. < 0.001 reference) and *Acacia* (0.009 ± 0.902 vs. < 0.001) were frequent in alternative sites (100% prevalence) but relatively absent from reference sites. *Pomaderris* was also more abundant in alternative sites (0.008 ± < 0.001 vs. < 0.001) and was a significant indicator genus (*p* < 0.05). Conversely, *Epacris* was more common in reference sites (0.088 ± 0.023 vs. 0.008 ± < 0.001 in alternative) occurring in 80% of sites and, as it only occurred in 10% of alternative sites, it was a strong reference state indicator.

Of the 16 species listed in the EVC benchmark, 12 were found in the reference communities and 10 in the alternative communities (Table S4). *E. obliqua* was more abundant than *E. baxteri* as the dominant overstorey species in reference sites and occurred sporadically in three alternative sites. Species not listed in the EVC but characteristic of the reference state surveyed in this study included an herbaceous climber *Billardiera macrantha*, the shrub species *Correa reflexa*, *Epacris impressa*, *Goodenia ovata*, *Spyridium parvifolium*, *Tetratheca cilliata*, *K. ambigua* and a species of small tree *Pomaderris aspera*.

### 3.2 Structural Diversity

#### 3.2.1 Diversity of Growth Forms

**FIGURE 3:**
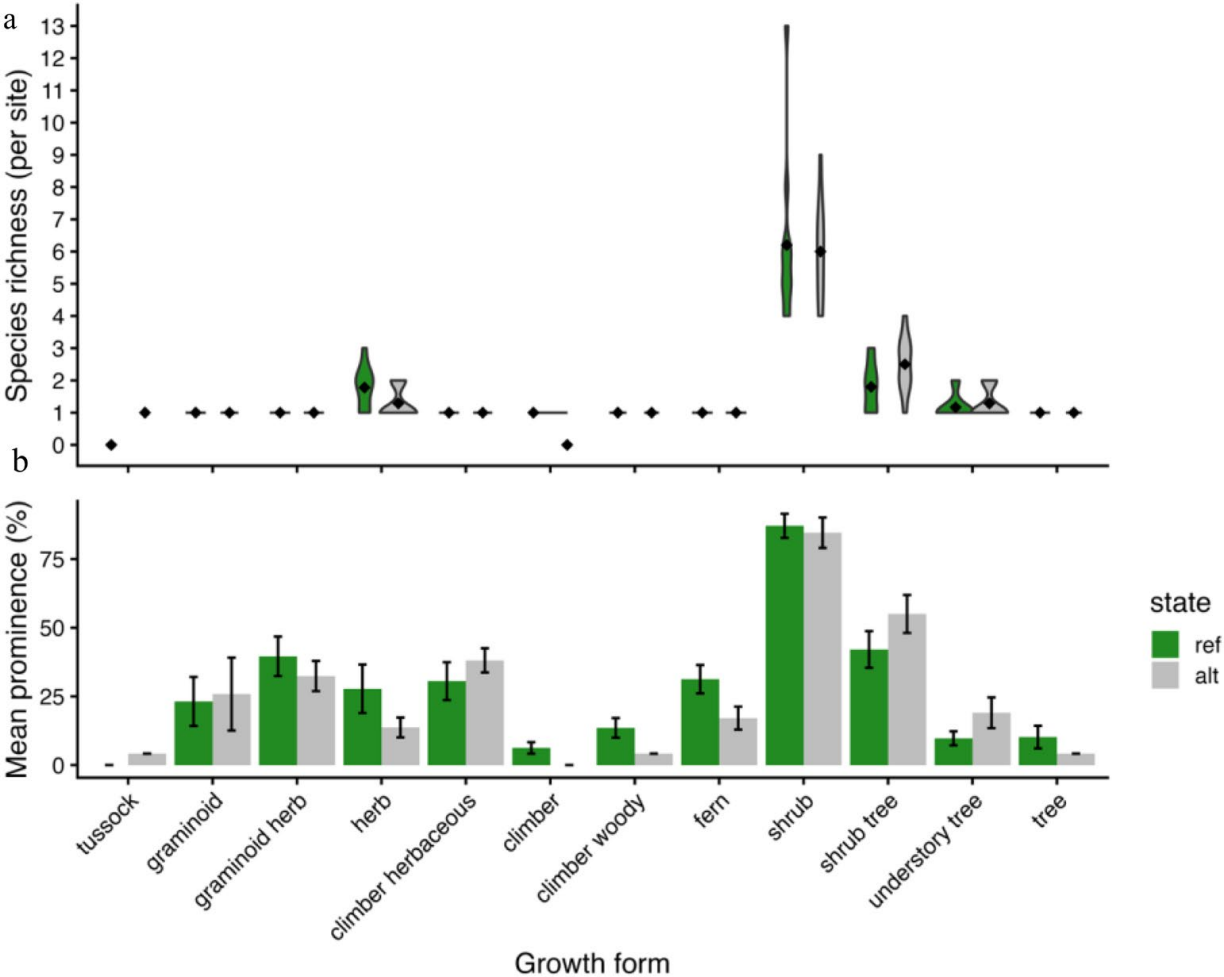
Comparison of the dominance of understorey growth forms between the alternative and reference states. (a) Violin plots of species count for each growth form by state (mean = ¨). (b) Comparisons of the mean prominence of growth forms between the two states. Prominence represents the percentage of sites the growth form occurred in, and error bars indicate standard error.

A total of 11 growth forms were counted across the alternative and reference states. On average, reference sites supported significantly more growth forms (9 ± 0.5 SE) than alternative sites (7 ± 0.4; *p* < 0.01). Growth form composition differed significantly between reference and alternative sites with state explaining ~17% of the growth form assemblage (*R* ^2^ = 0.17, *p* < 0.05; Figure 2b). Trees (e.g., *Eucalyptus*) were a significant indicator growth form in reference forests (0.004 alternative vs. 0.026 reference; *p* < 0.05; Figure 3b).

#### 3.2.2 Structural Diversity of Standing Stems

**FIGURE 4:**
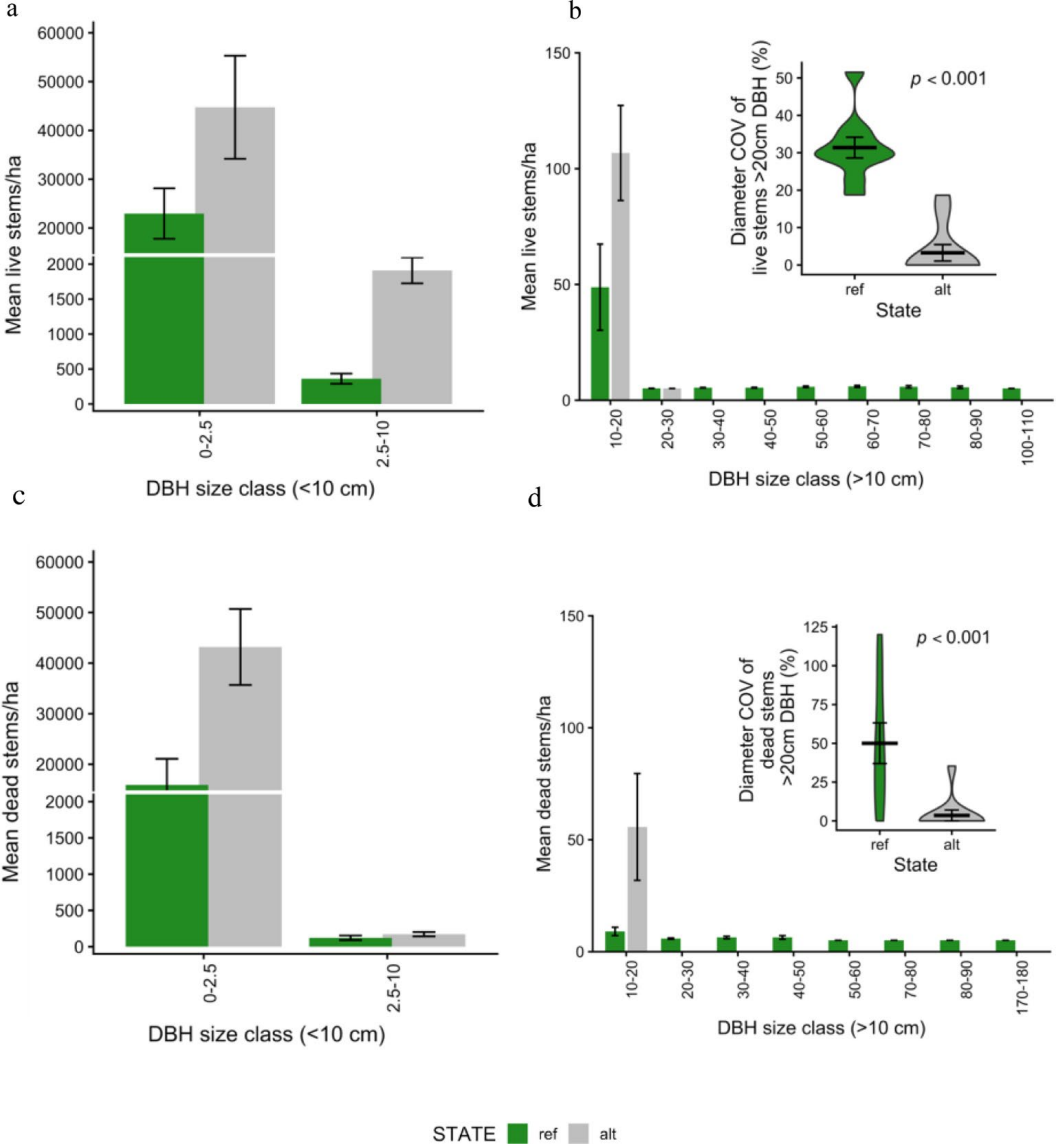
Distribution of live (a) and dead (b) stems per hectare (stems/ha) across different forest states. Error bars indicate the standard error. Stems with a diameter < 10 cm are grouped into size bins based on survey method; stems ≥ 10 cm are displayed in 10 cm diameter intervals. The top‐right inset (c) violin plots display the coefficient of variation (COV) of live stem diameters in the largest size class (DBH > 20 cm), and the bottom‐right inset (d) the COV of dead stems in the same size class.

Forests in an alternative state were dominated by stems in smaller size classes (< 20 cm DBH; Figure 4a,c) and total stem density was 123% higher in alternative sites (87,959 ± 12,207 SE) than reference sites (39,413 ± 10,136; *p* < 0.05). Weighted mean stem diameter was also lower in alternative sites (15.1 ± 3.06) relative to reference sites (23.4 ± 0.49; *p* < 0.05), with a distinct lack of trees > 20 cm diameter in alternative sites (5.09 ± 3.72 alternative vs. 291 ± 35 reference; *p* < 0.05). The coefficient of variation (COV) indicated greater uniformity of live and dead stem sizes at alternative sites (80.7 ± 2.83) relative to the reference sites (202 ± 21; *p* < 0.001). Of the large trees, stems were on average 87% smaller in the alternative sites (4.76 cm ± 3.18) compared to reference sites (39.1 cm ± 3.64, *p* < 0.001). The COV of live stems was 89% lower in alternative states (Figure 4b: 3.26 ± 2.20 alternative vs. 31.4 ± 2.77 reference; *p* < 0.001) and the dead stems 93% lower (Figure 4d; 3.54 cm ± 3.54 vs. 50 cm ± 13.1; *p* < 0.001).

#### 3.2.3 Structural Diversity of Coarse Woody Debris

**FIGURE 5:**
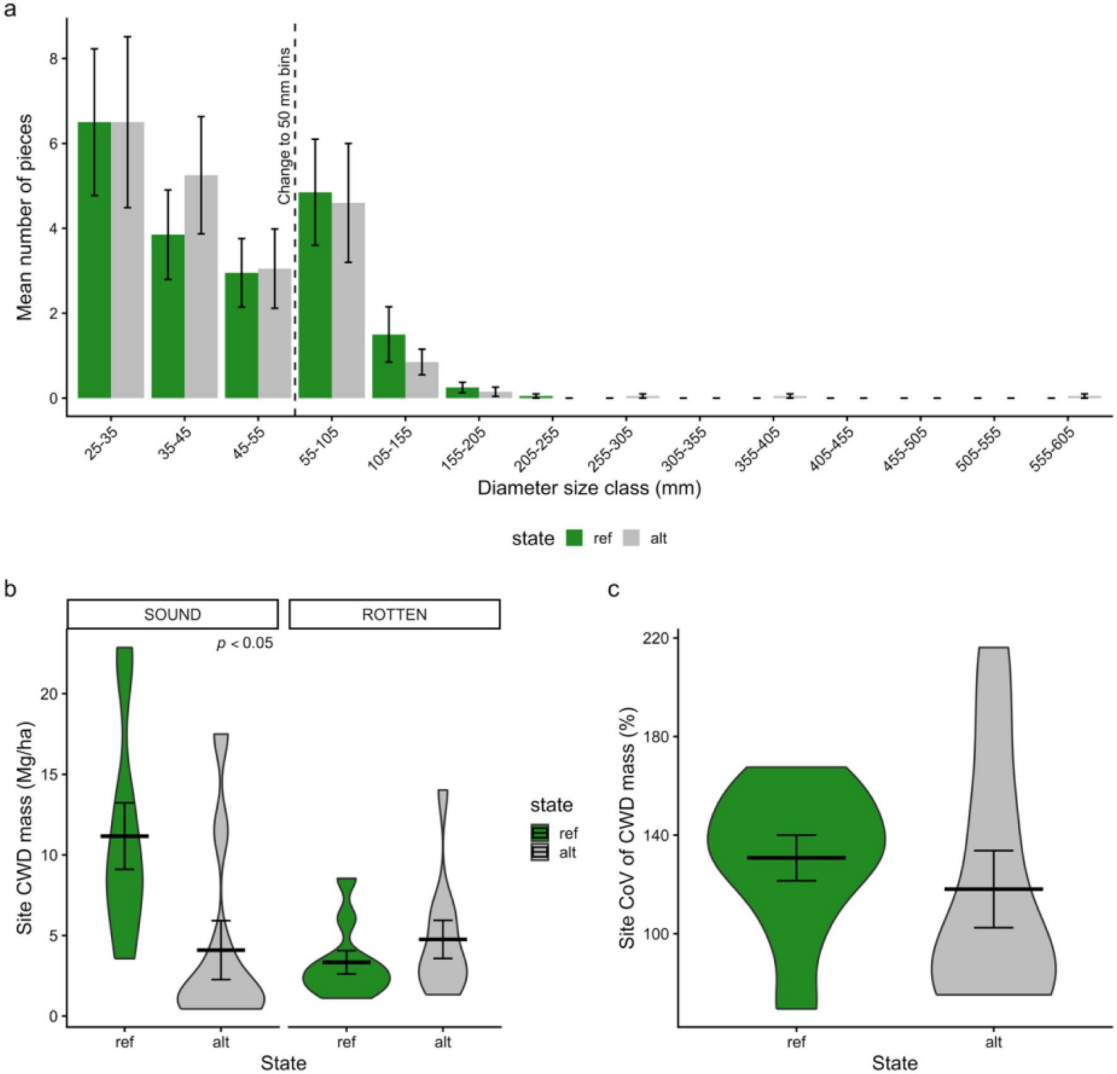
(a) Frequency of coarse woody debris (CWD) by reference and alternative states. Note that the *x* axis is not continuous bin sizes with the change indicated by the dashed line (10 mm bin size ranging from 25 to 55 mm and 50 mm size classes from 55 to 605 mm). The error bars depict standard error. (b) Violin plots compare the mass of sound and rotten CWD (Mg/ha) between states. Mean is indicated by the bold centreline, and the error bars indicate the standard error.

Alternative sites supported 38% lower CWD mass compared to reference sites (14.5 ± 2.43 Mg/ha reference vs. 8.86 ± 1.69 Mg/ha alternative; *p* < 0.05). Frequency of CWD pieces by size class was comparable between states, except for the largest size classes (> 295 mm), which were absent from the reference sites (Figure 5a). The variability of CWD piece size as indicated by the mean COV of piece mass (Figure 5c) was alternative 118 Mg/ha (±15.6) in alternative sites and 131 Mg/ha (±9.26) in reference states but not statistically significant. Mass of sound CWD was significantly greater in reference (21.9 ± 2.07 Mg/ha) than alternative states (7.98 ± 1.83; *p* < 0.05), but there was no significant difference in the mass of rotten CWD between states (Figure 5b).

#### 3.2.4 Structural Complexity and Site Dissimilarity

There were no statistical differences in overall structural complexity between alternative and reference states. Nonetheless, there were significant differences in cover by stratum. The cover of all stratum between 20 cm and the canopy layer was greater in the alternative than reference states, and the 100–200 cm strata significantly higher (0.06% ± 0.08% ref vs. 0.8 ± 0.04 alt; *p* < 0.05; Figure S5). Conversely, the mean cover of the ground strata (0–20 cm category) was significantly lower in the alternative (61% ± 0.05) than reference states (92% ± 0.08; *p* < 0.001). Multivariate analyses indicated significant differences in vegetation dissimilarity between states. State explained ~18.7% of the variation in structural dissimilarity (*p* < 0.05), while the ANOSIM also detected moderate but significant separation (*R* = 0.238, *p* < 0.05). Alternative sites were characterised by greater structural homogeneity in mid‐storey vegetation (20–200 cm stratum) relative to reference sites. While reference sites showed greater homogeneity in the canopy and more consistent ground cover (0–20 cm). PC1 captured the dominant gradient in vegetation structure, explaining 28.9% of variance, and contrasted sites with high dissimilarity in the mid–upper vegetation strata (20–200 cm; negative scores) against those with higher dissimilarity in canopy and ground‐layer structure (0–20 cm, litter depth, overstorey cover; positive scores). Site‐level PC1 scores differed significantly between states (*p* < 0.05) consistent with the dissimilarity‐based tests.

### 3.3 Ecosystem Function

#### 3.3.1 Carbon Storage

**FIGURE 6:**
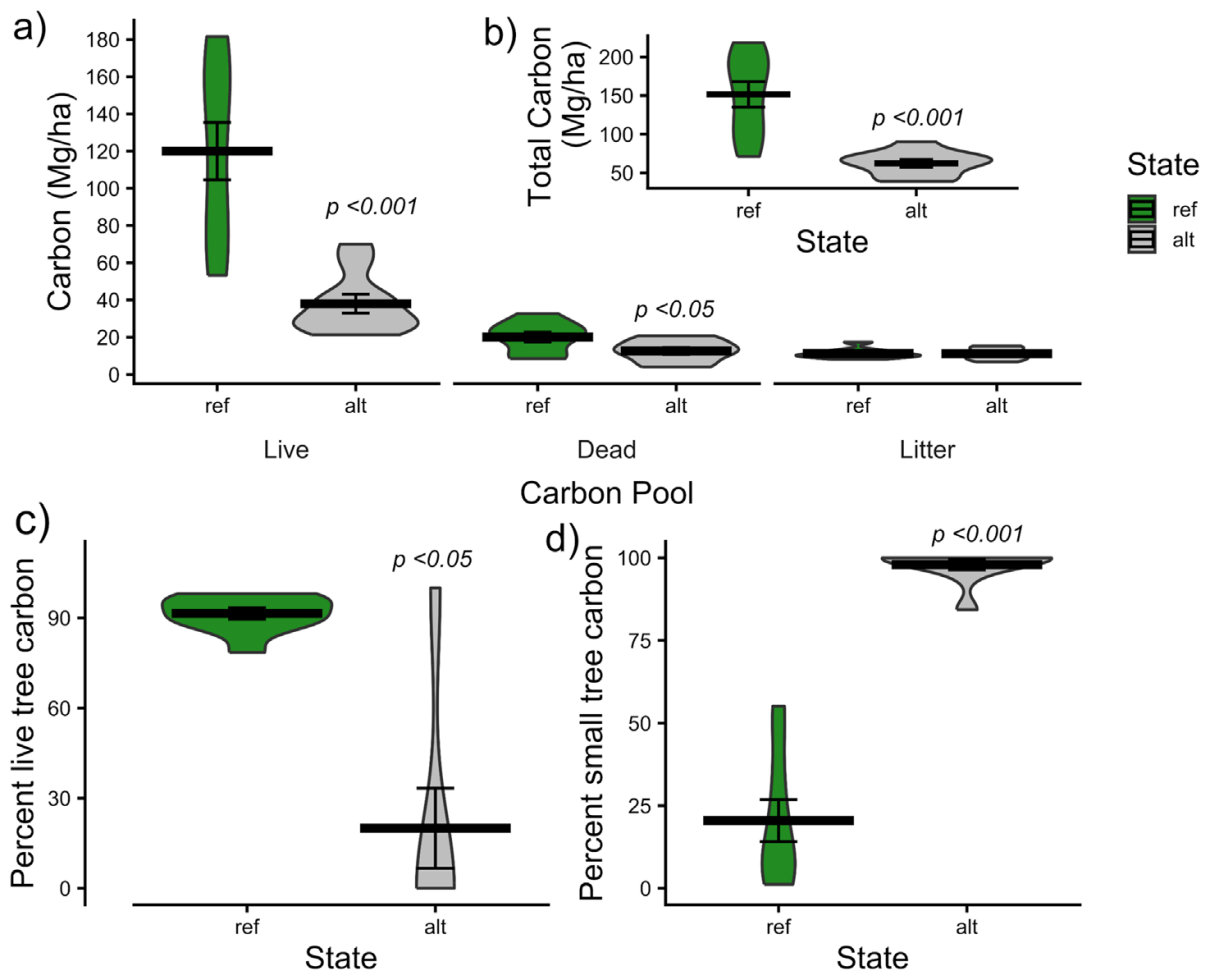
(a) Violin plots indicate that reference sites stored significantly greater total carbon than alternative sites (*p* < 0.05). The bold line indicates the mean and error bars are the standard error. (b) Reference sites stored more carbon in the live tree, and dead pools (*p* < 0.05). (c) Percentage live tree carbon (d) Percent small tree carbon (trees < 20 cm DBH).

Alternative sites stored ~40% less total above‐ground carbon than reference sites (62.2 Mg/ha ± 4.98 alternative vs. 152 ± 16.6 reference *p* < 0.001; Figure 6b). This difference was most pronounced in the live tree carbon pool where alternative sites stored 38 Mg/ha (±5.0; Figure 6a) compared to 120 Mg/ha (±15.4; *p* < 0.001) in reference forests. Carbon stocks in the dead carbon pool were also significantly lower in alternative sites (12.7 ± 1.8 Mg/ha vs. 20.1 ± 2.6 reference: *p* < 0.05), whereas differences in the litter pool were non‐significant. Percent live tree carbon was 78% lower in alternative sites (20.5% ± 13.9) relative to reference sites (91.5% ± 1.91; *p* < 0.05; Figure 6c). Close to all live tree carbon 98% (±1.59) was stored in small trees in alternative sites compared to ~20% (±6.37; *p* < 0.001) in reference sites.

#### 3.3.2 Probability of Hollows

**FIGURE 7:**
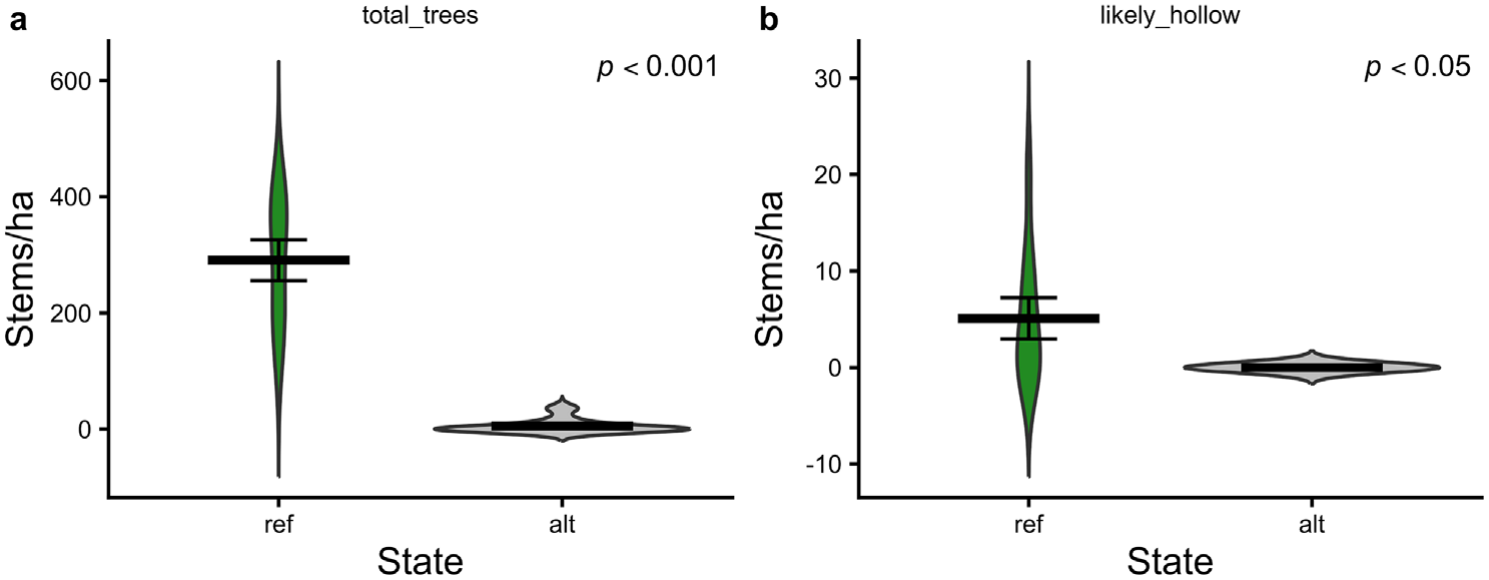
(a) Mean number of trees/ha > 20 cm DBH and (b) mean number of trees with a > 50% likelihood of supporting tree hollows of reference and alternative states. The mean and standard error are indicated by the crossbars.

Forests in an alternative state typically lacked trees capable of supporting hollow formation (*p* < 0.001). In reference states, of 291 (±35) stems per hectare (*p* < 0.001; Figure 7a), on average 5 (±2.15) had a > 0.5 probability of containing a hollow (Figure 7b). In contrast, alternative states supported just 6 (±3.71) stems/ha, none of which were predicted to support hollows (*p* < 0.05). Hollow formation was more likely to occur at smaller diameters in dead trees than live trees and the thresholds of 0.5 probability of hollow presence varied among species. *Eucalyptus baxteri* and *E. obliqua* were estimated to reach a 50% probability of hollow formation at DBHs of 94.2 and 92.0 cm, respectively. In contrast, standing dead trees reached this threshold at 57.3 cm DBH.

#### 3.3.3 Seed Dispersal and Pollination Strategies

The number of plant species requiring invertebrates or vertebrates for pollination or seed dispersal did not differ significantly between states. Of the 42 plant species identified in the study, only four were pollinated by either invertebrates or vertebrates. On average, there were 2.67 (±0.33) of these plants in alternative states and 2.56 (±0.34) in the reference forest (Figure S6). Invertebrates were the key dispersal agent of seeds, with an average of 6.5 (±0.56) dispersed by invertebrates in alternative states and 7.7 (±0.67) in reference sites. The number of plant species dispersed by vertebrates was 1.4 (±0.22) in alternative versus 1.5 (±0.27) in reference forests.

## 4 Discussion

Repeated short‐interval fires occurring between 1939 and 1951 shifted temperate eucalypt forests into alternative states with altered structure, composition, and function that persisted for over 74 years and through subsequent fires in 2005 and 2009. Measurements taken 16 to 20 years after the most recent fire highlighted a near‐absence of large trees in the alternative state, with substantial consequences for carbon storage and habitat provision. Reductions in coarse woody debris and compositional changes in the understorey and mid‐storey have implications for biodiversity, fuel structure, microclimate, and hydrology. Active intervention may be required under emerging fire regimes. Our empirical comparisons can guide prioritisation of management beyond more qualitative comparisons with reference benchmarks.

### 4.1 Compositional Shift

*Eucalyptus* was absent from 70% of the alternative sites and, where present, occurred in substantially lower densities compared to the reference state. Large trees such as *Eucalyptus* are keystone structures that maintain forest processes including carbon storage (Keith et al. 2009), habitat provisioning (Remm and Lõhmus 2011), and influence hydrology, nutrient cycling, and microclimate conditions (Lutz et al. 2018). *Eucalyptus* are characteristic of the overstorey in most temperate Australian forest types and a defining feature of ecosystems as forest (Costermans 2009; Specht 1970). Furthermore, the species, size, and density of *Eucalyptus* are used to assess forest condition against benchmarks of ecosystem quality (DEECA 2025). Decreased numbers of resprouting *Eucalyptus* following short‐interval fires have been reported in similar dry sclerophyll forests (Fairman et al. 2022). In this study, absence of a tall canopy in the alternative state suggests they no longer meet the structural criteria of a forest (Specht 1970). The measured shift to shorter simplified vegetation in our study is consistent with the findings of Heap et al. (2025) and Ashton and Chappill (1989). Five years after the last fire, Heap et al. (2025) describe the structure of the alternative state in this system as consistent with shrubland/scrub (mean height 5 m, canopy cover 7%). However, our field‐based assessments 19 years after the last fire in 2005 indicate the alternative state is more characteristic of a low closed forest (mean height 5 m, canopy cover 72%; Specht 1970).

Understorey composition differed significantly between the states. This was driven by several genera associated with alternative sites such as *Billardiera*, *Kunzea*, *Acacia*, *Pomaderris* and driven by the significant lack of small trees. *K. ambigua* was not characteristic of the EVC benchmark and yet occurred in 100% of alternative sites compared to 30% of reference sites. Proliferation and dominance of this native shrub after fire may be due to traits that confer competitive advantage, such as rapid establishment and effective competition through shading (Ashton and Chappill 1989; Judd 1990; Morgan and Nield 2011).

### 4.2 Structural Simplification

Stem density at alternative sites was more than twice that of reference forests, with most stems in the 0–20 cm DBH classes. This likely reflects the competitive release and change in dominance to understorey trees, shrub‐trees, and shrub growth forms with thinner stems (Leuchner et al. 2012). This also meant vegetation cover in the 100–200 cm strata was significantly greater in alternative sites. Consistent with our findings, increased density of the mid‐storey was also reported in studies of alternative states in temperate eucalypt forests (Brown et al. 2021; Burton et al. 2019). Conversely, cover in the 0–20 cm strata was consistently lower in the alternative than reference state due to less occurrence of the herb growth form.

Alternative sites were structurally more homogenous than reference forests, particularly in the mid‐storey vegetation. This was reflected in lower coefficients of variation for stem DBH and large tree DBH (> 20 cm DBH) and lower values of dissimilarity indicated in the PCA. Structural diversity is closely linked to ecosystem function and biodiversity through niche partitioning (LaRue et al. 2023; MacArthur and MacArthur 1961; Stein et al. 2014). For example, structural diversity has been linked to greater carbon storage in temperate *Eucalyptus* forests due to utilisation of space and therefore maximisation of resources (Aponte et al. 2020). The overall structural complexity index did not differ significantly between states, despite variability in stem size and significant differences in cover of the 0–50 cm and 100–200 cm height stratum. This index accounts for canopy‐to‐ground vegetation cover and litter depth (Swan et al. 2015). While it suggests retained complexity, differences between strata outlined above can have species‐specific outcomes. For example, increased consistent mid‐storey cover in the alternative sites could be favourable to small mammals such as long‐nosed potoroo ( *Potorous tridactylus* ), long‐nosed bandicoot ( *Perameles nasuta* ), and Australian swamp rat ( *Rattus lutreolus* ) (Swan et al. 2015). Thus, while overall complexity is maintained, habitat value may still be functionally altered.

Mass of coarse woody debris (CWD) was considerably lower in alternative than reference sites. The amount of CWD in a forest is generally reflective of the size and density of the dominant tree species with large tree species a major contributor to CWD inputs (Harmon et al. 1986; Woldendorp and Keenan 2005), typically replenishing large amounts of CWD stocks post‐fire (Bassett et al. 2015). While CWD mass in reference forests was similar to the average CWD mass reported for open *Eucalyptus* forests (Burton et al. 2021), the lower mass in alternative sites reflects an absence of large trees and associated transfers to fallen wood (Harmon et al. 1986). The absence of large standing stems and the comparatively lower mass of sound CWD in alternative states indicates that recruitment of additional large pieces has not and will not occur for some time. Large pieces of CWD (253–605 mm diameter) at alternative sites reflected the legacy of past disturbances and contributed to a similar mass of rotten CWD between states. The amount and decomposition rates of CWD varies with topographic position, fire regime and microclimate conditions (Bassett et al. 2015; Grove and Meggs 2003; Mackensen et al. 2003). However, without ongoing inputs, continued decay will gradually deplete the CWD pool, reducing the diversity of sizes and decay classes and potentially leading to its near absence in alternative‐state sites, thereby limiting associated biodiversity support (Grove and Meggs 2003).

### 4.3 Functional Implications

Canopy loss in the alternative state has significantly impacted several ecosystem functions. Our findings underscore the vulnerable nature of carbon pools in the alternative state, where the dominance of small, fire‐vulnerable stems compromises long‐term carbon stability. Forests in an alternative state stored significantly less total carbon across all the above‐ground pools—including the live carbon pool—than the reference state. As the live carbon pool provides the active carbon sequestration, the conversion of forests from a reference to an alternative state may reduce the sequestration potential of this landscape (Stephenson et al. 2014). In both states, the live carbon pool stored the greatest amount of carbon. Resistance to disturbances such as fire comes with increasing tree size and increases the likelihood of post‐fire recovery (Brando et al. 2012; Nolan et al. 2021). Greater carbon stability in resprouter forests is associated with a higher proportion of carbon stored in large living trees (L. T. Bennett et al. 2017). More carbon stored in small live stems in the alternative state makes that carbon more vulnerable to decreases through tree mortality (i.e., transfers to the deadwood pool) and subsequent decomposition or combustion in later wildfires (Bennett et al. 2017; Fairman et al. 2022; Lawes et al. 2011). In combination, decreased carbon sequestration potential and increased vulnerability of live tree carbon to subsequent fires means that the alternative state will be a less reliable carbon sink than the reference state (Bennett et al. 2016, 2017; Brown and Johnstone 2011).

Coarse woody debris and large trees play a vital role in habitat provisioning. Hollow‐bearing trees are critical to many Australian vertebrates, including microbats, arboreal mammals, and birds (Bennett et al. 1991; Gibbons and Lindenmayer 2002; Goldingay 2009; Lindenmayer et al. 2012; Penton et al. 2021; Wagner et al. 2024). Hollow formation can take decades and is influenced by stem size, fire exposure, and environmental factors (Koch et al. 2008; McLean et al. 2015). In the alternative state, hollow‐bearing trees were entirely absent, and given the smaller‐diameter profile of remaining stems, hollow recruitment in coming decades will be minimal. The shift to a structurally simplified forest with fewer large trees may reduce faunal diversity and increase competition for remaining habitat structures (Baroni et al. 2020; Brockerhoff et al. 2017; Goldingay 2009; Lindenmayer and Laurance 2017). CWD is also an important component of forest structure. It provides habitat for a range of organisms from small vertebrates to invertebrates and fungi while contributing to nutrient cycling (Bässler et al. 2010; Grove and Meggs 2003; Mac Nally et al. 2001). Consequently, the reduced availability of CWD and hollow‐bearing trees in the alternative state exacerbates the decline in habitat provisioning.

Structural changes in cover and density can be used to infer changes to microclimate regulation and hydrological processes. Forest structure influences wind speed, radiation interception, and evaporation (De Frenne et al. 2019; Mwamulima et al. 2025; Zellweger et al. 2019). Less canopy and increased mid‐storey cover in the alternative state could produce cooler, damper ground conditions due to reduced radiation and wind speeds. Greater canopy closure and stem density have been associated with increased microclimate buffering by reducing maximum temperatures and vapour pressure deficit in temperate *Eucalyptus* forests of south‐eastern Australia (Brown et al. 2021; Burton et al. 2021; Mwamulima et al. 2025). In terms of hydrology, studies in similar systems have shown that changes in canopy composition can reduce transpiration and increase streamflow (Lakmali et al. 2022, 2023; Robinne et al. 2018). These findings imply potential water yield increases from the alternative state, although further research is needed for dry sclerophyll forests.

Despite concerns that altered species composition could reduce resources for pollinators and dispersers, our results suggest limited differences in the prevalence of biotically dispersed or pollinated species between forest states. Flora that rely on biotic interactions such as pollination and seed dispersal often evolve mutualistic relationships with fauna to facilitate these processes (Bronstein et al. 2006; Hagan and Grove 1999; Mitchell et al. 2009). Consequently, shifts in species composition that reduce the availability of floral resources could compromise resource provisioning within the landscape, diminishing the likelihood of attracting fauna capable of dispersing seeds into these sites. It has been proposed that resources available to pollinators and dispersers may be reduced in acacia‐dominated shrublands (Lavorel et al. 2015). However, the results of our study indicated no difference in the number of plant species requiring pollination or seed dispersal via biotic processes between states. Although not statistically significant, the reduction in plant species requiring zoochorous seed dispersal may limit the introduction of absent tree species to the alternative state, compounding shifts in composition (Bueno et al. 2013; Lehouck et al. 2009; Liss et al. 2013). However, these changes in resources may be considered moderate in this landscape and therefore may not be a key concern to land managers.

### 4.4 Management Implications

Our study identified specific areas of ecological degradation and functional loss due to the conversion of forests to an alternative state by applying several of the ecosystem attributes outlined in the Standards for the Practice of Ecological Restoration (Gann et al. 2019). While ecosystem functions refer to the biophysical processes that occur in nature regardless of human benefit, ecosystem services frame those functions through a human‐centric lens (Brockerhoff et al. 2017; Costanza 1997; Costanza et al. 2017; de Groot et al. 2010). Changes in ecosystem function in this study have implications for the sustainable delivery and management of ecosystem services, such as carbon sequestration, biodiversity support (habitat provisioning), microclimate regulation, water supply, and underpin the need for management strategies that anticipate future landscape transitions (Costanza et al. 2017; de Groot et al. 2010). Ecosystem services provide a useful framework for aligning data on degraded ecological function with societal values and benefits, including impacts on policy and economic planning (Birgé 2016; Costanza 1997).

Not included in our assessment is the effect of state change on disturbance regulation (Costanza et al. 2017), particularly relating to fire. Positive feedbacks have been recorded in forests converted to shrub‐dominated systems where the vegetation shift has increased the likelihood and severity of subsequent fires. This process favours reestablishment of pyrophilic species and leads to the exclusion of fire‐sensitive species in the landscape (Coop et al. 2020; Tepley et al. 2018). How this positive feedback loop might play out in our alternative state remains unknown. However, greater height and cover of the 100–200‐cm stratum (elevated fuels) have been associated with increased flame height and fire line intensity in eucalypt‐dominated forests (Gould et al. 2008). Therefore, relatively greater height and cover of elevated fuels in our study's alternative state might result in more intense fire behaviour. Conversely, ground cover (near surface fuel) was lower and less continuous in the alternative than reference state and could lead to decreases in the rate of spread in the former (Gould et al. 2008). CWD mass can also influence fire behaviour and severity, with greater levels of decay related to ignition success and greater consumption rates (Hollis et al. 2011, 2018). While sound CWD was more abundant in reference states, there was minimal difference in the mass of rotten CWD between states. Proportionally more CWD in rotten than sound classes could indicate greater relative vulnerability to consumption in subsequent fires at the alternative sites. With changing fire regimes in southeastern Australia predicted to include more short‐interval fires, land managers will need to anticipate an increased likelihood of alternative forest states (Abram et al. 2021; Kelly et al. 2023; Nolan et al. 2021). Understanding the potential changes in fire behaviour and landscape flammability is essential for predicting long‐term landscape resilience in fire‐prone temperate Australia, and to informing management strategies aimed at mitigating fire risk and preserving ecosystem function.

The longevity and stability of this study's alternative sites, along with minimal *Eucalyptus* recruitment, suggest that passive recovery to reference conditions is unlikely without intervention. Our results indicate a need for active restoration to overcome biotic thresholds. *Eucalyptus* regeneration was generally absent in the alternative state, except for one site where there were a few *Eucalyptus* seedlings after the 2005 fire near an ecotone boundary. This absence of *Eucalyptus* regeneration is consistent with ecological constraints of the species, including limited dispersal distances (typically up to the parent tree height) and requirements for a clear, competition‐free seedbed to establish (Booth 2017; Bowd et al. 2023). Furthermore, a fire‐free period of at least 15 to 20 years would be needed for individual eucalypts to reach reproductive maturity (Bowd et al. 2023; Fairman et al. 2016; Nolan et al. 2021). The time required for eucalypt stems to grow to a ‘large’ size (> 20 cm DBH) that is sufficient to survive most fires through epicormic resprouting (Fairman et al. 2019) will likely be even longer, although this is difficult to predict given the highly variable and opportunistic growth of eucalypts in mixed‐species stands (Hinko‐Najera et al. 2019; Plumanns‐Pouton et al. 2022).

Active restoration is expensive, labour‐intensive, and requires costly activities such as, aerial seeding—a technique used to reintroduce key overstorey species in other degraded *Eucalyptus* ecosystems (Bassett et al. 2015). When funding is limited or restricted to funding cycles, strategic decisions must be made about where to invest resources due to the complexity and scale of active intervention in this topographically complex landscape. Therefore, restoration strategies could benefit from the adoption of operational decision‐making frameworks (Lynch et al. 2021; Meyer et al. 2021). In an era of rapid ecosystem transformation, these frameworks assist managers to strategically prioritise actions for wildlife, natural resources and ecosystem function. A prominent example is the Resist–Accept–Direct (RAD) framework for managing ecosystem transformation (Copenhaver‐Parry and Byerly 2025; Lynch et al. 2021; Scholtz et al. 2025). Within this framework, a resist strategy seeks to prevent transformation by maintaining historical ecosystem conditions. This may involve restoring the composition, structure, and function of a reference state (Gann et al. 2019). Or the protection of areas at high risk of conversion to alternative states (e.g., recently burned by short‐interval wildfires), existing forests of high ecological value, such as climate and/or wildfire refugia (Meddens et al. 2018), or long‐unburnt forests (Keenan and Read 2012; Lindenmayer and Bowd 2022). Alternatively, under an accept pathway, managers would not aim to restore forests to reference conditions but instead allow an alternative state to persist under minimal intervention. Finally, using the direct approach, managers accept that the system will not return to its historical condition but intervene to influence the trajectory of change towards a preferred alternative state (Lynch et al. 2021). Within a direct management pathway, managers might introduce a surrogate canopy‐forming species or a more fire‐resilient *Eucalyptus* species to re‐establish the large‐tree structural component in anticipation of future fire regimes in our study area (Gann et al. 2019; Lynch et al. 2021). However, each RAD pathway offers trade‐offs among management objectives, societal values, and available resources, and must consider the pace and magnitude of ecosystem change, which can be highly uncertain.

Our results also highlight the limitations of using generalised ecosystem benchmarks such as Ecological Vegetation Classes (EVCs) to assess condition and define a single reference state. In the Shrubby Foothill Forest examined at our sites, there were several species that were not included in the EVC list. Our approach assesses the integrity of broader ecosystem processes and resilience. This aligns with the acknowledged need to consider and quantify functions and services in restoration ecology and ecological restoration decision‐making (Aronson et al. 2017; Stanturf et al. 2014). However, such measures remain under‐reported in the literature (Kollmann et al. 2016; Montoya et al. 2012; Shimamoto et al. 2018), highlighting the contribution of this study as an example of multiple functional evaluations to improve operational understanding of ecosystem state changes.

## 5 Conclusions

Repeated short‐interval fires can drive persistent state changes in fire‐tolerant eucalypt forests of temperate Australia. These shifts are associated with measurable changes in ecosystem structure, function, and the provision of key ecosystem services such as carbon sequestration, habitat provisioning and biodiversity support. Given the persistence and extent of these changes, passive recovery appears unlikely. Therefore, restoration in these systems will require active intervention. To inform and prioritise efforts, we quantified the attributes of alternative sites and compared them to reference forests. We provide empirical measures that quantify structural and functional divergence between the alternative and reference states and provide a practical foundation for restoration planning. By measuring the degree of structural and functional divergence, these data offer a strategic and evidence‐based foundation for restoration planning and forest management under changing disturbance regimes. Future research should extend this approach to other ecosystems and integrate ecological data with predictive fire models. Doing so will help managers identify priority restoration sites with lower fire risk, an urgent need as climate change amplifies disturbance pressures on multiple ecosystems in fire‐prone landscapes.

## Author Contributions

**Lauren T. Bennett:** conceptualization, writing – review and editing, methodology, supervision, resources, investigation, formal analysis. **Aaron E. Heap:** conceptualization, investigation, funding acquisition, writing – original draft, methodology, validation, visualization, writing – review and editing, software, formal analysis, project administration, resources, supervision, data curation. **Tom A. Fairman:** conceptualization, funding acquisition, methodology, writing – review and editing, project administration, supervision. **Trent D. Penman:** conceptualization, funding acquisition, writing – review and editing, methodology, resources, supervision.

## Funding

Australian Government Research Training Program Scholarship (https://doi.org/10.82133/C42F‐K220) awarded to Aaron E. Heap (PhD candidate); Parks Victoria Research Partnerships Program; the Friends of Wilsons Promontory; Victorian Environmental Assessment Councils' Bill Borthwick Student Scholarships; Samuel Austin Frank Pond Traveling Scholarship.

## Conflicts of Interest

The authors declare no conflicts of interest.

## Supporting information


**Appendix S1:** gcb70895‐sup‐0001‐Supinfo.zip.

## Data Availability

The data that support the findings of this study are openly available at Figshare: https://doi.org/10.26188/30546542.
