# Supplementary material for: Characterising Ecosystem Composition, Structure and Function of Alternative Stable States in Temperate Forests of South‐Eastern Australia
Source: Glob Chang Biol. 2026 May 5;32:e70895. doi: 10.1111/gcb.70895 (PMC13140778; doi:10.1111/gcb.70895)

**Supplementary Material**

Figure S1- Diagram of the plot layout and nested plots, transects and litter sampling locations


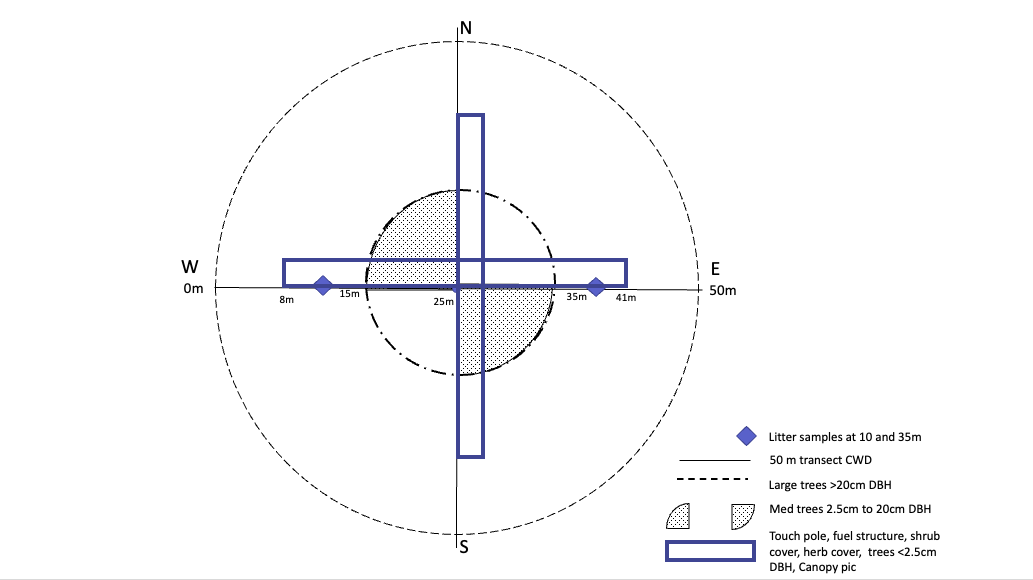


TABLE S2. Allometric equations used in the calculation of carbon stocks. The

independent variable most cases was diameter breast height over-bark (DBH), with shrub equations based on % foliage projective cover.

| Dependent variable | Species-specific or general | Equation form ^c^ | Reference |
| --- | --- | --- | --- |
| Large tree biomass  (> 50 cm DBH) ^a^ | general | Logistic | (Dean *et al.*, 2012) |
| Tree biomass  (20 to 50 cm DBH) ^a^ |  |  |  |
| *E. baxteri* | species | Log-Log | (Bi *et al.*, 2015) or (Keith *et al.*, 2000) |
| *E. obliqua* | species | Log-Log | (Keith *et al.*, 2000) |
| *Eucalyptus hybrid - E. obliqua* | species | Log-Log | (Keith *et al.*, 2000) |
| *Eucalyptus* (species unknown) | general | Log-Log | (Keith *et al.*, 2000) |
| All other tree species ^b^ | general | Logistic | Understorey tree species equation, (Dean *et al.*, 2012) |
| Tree biomass  (<20 cm DBH) | general | Logistic | Understorey tree species equation, (Dean *et al.*, 2012) |
| Shrub Biomass |  |  |  |
| Medium shrubs | General | Linear | Acacia shrubland equation (Grierson et al., 1992) |
| Short/prostate shrubs | General | Linear | Native chenopod equation (Grierson et al., 1992) |

^a^ Used irrespective of species for all stems >50 cm DBH, also used in a weighted average with the below assigned allometric equations for estimations in the DBH range 35 to 50 cm

S3 - Hollow modelling formula

For each species, we modelled hollow presence using a log-logistic regression:

$$Y\sim\text{Bernoulli}\left( \frac{1}{1+\exp\left[ -\left( \alpha+\beta\log\left( \text{DBH} \right) \right) \right]} \right)$$

Where Y is a binary observation for hollow presence (0 = absent, 1 = present), p is the modelled probability of hollow presence, a is the intercept of the model on the logit scale, and b is the slope of the model on the logit scale.

TABLE S3a: summary statistics of the data used to develop hollow-DBH allometries

| Species/tree status | # plots | # trees | mean DBH (cm) | min DBH (cm) | max DBH (cm) |
| --- | --- | --- | --- | --- | --- |
| Eucalyptus baxteri | 54 | 249 | 57.6 | 20.7 | 142.2 |
| Eucalyptus obliqua | 526 | 4018 | 79.2 | 20.0 | 315.0 |
| Standing dead tree | 799 | 1806 | 81.6 | 20.0 | 353.0 |

TABLE S3b: Coefficient summary

|  | Intercept (a) | | Slope (b) | |
| --- | --- | --- | --- | --- |
| Species/tree status | Mean | SD | Mean | SD |
| Eucalyptus baxteri | -15.23 | 2.46 | 3.35 | 0.58 |
| Eucalyptus obliqua | -21.07 | 0.60 | 4.66 | 0.13 |
| Standing dead tree | -17.41 | 0.70 | 4.30 | 0.17 |

TABLE S4: Comparison of number of occurrences of plant species across sites in reference (n=10) and alternative (n=10) states. Species listed in the Ecological Vegetation Class benchmark for Shrubby Foothill forests are indicated by *. Species growth forms were retrieved from the AusTraits database and missing values were supplemented from VicFlora database (Falster et al., 2021).

| Growth Form*^A^* | Species | Total Occurrences Across Reference Sites (n=10) | Total Occurrences Across Alternative Sites (n=10) |
| --- | --- | --- | --- |
| Graminoid | *Lepidosperma laterale** | 9 | 5 |
| Graminoid herb | *Tetrarrhena juncea** | 10 | 9 |
| Herb | *Viola hederacea** | 2 | 1 |
| Herb | *Gonocarpus humilis** | 9 | 5 |
| Fern | *Pteridium esculentum** | 10 | 10 |
| Shrub | *Banksia spinulosa** | 3 | 7 |
| Shrub | *Bauera rubioides** | 1 | 0 |
| Shrub tree | *Zieria arborescens** | 4 | 8 |
| Shrub tree | *Acacia verticillate** | 10 | 10 |
| Understory tree | *Elaeocarpus reticulatus** | 3 | 2 |
| Tree | *Eucalyptus baxteri** | 1 | 0 |
| Tree | *Eucalyptus obliqua** | 9 | 3 |
| Tussock | *Lomandra filiformis* | 0 | 1 |
| Herb | *Amperea xiphoclada* | 4 | 2 |
| Herb | *Cyrtostylis reniformis* | 0 | 1 |
| Herb | *Dichondra repens* | 1 | 0 |
| Climber herbaceous | *Billardiera mutabilis* | 2 | 4 |
| Climber herbaceous | *Pandorea pandorana* | 1 | 0 |
| Climber | *Comesperma volubile* | 4 | 0 |
| Climber woody | *Clematis aristata* | 4 | 2 |
| Shrub | *Billardiera macrantha* | 8 | 7 |
| Shrub | *Correa reflexa* | 8 | 5 |
| Shrub | *Dillwynia sericea* | 1 | 0 |
| Shrub | *Epacris impressa* | 9 | 3 |
| Shrub | *Goodenia ovata* | 9 | 7 |
| Shrub | *Hakea sericea* | 0 | 1 |
| Shrub | *Isopogon ceratophyllus* | 1 | 0 |
| Shrub | *Kunzea ambigua* | 3 | 10 |
| Shrub | *Leptospermum continentale* | 0 | 2 |
| Shrub | *Leptospermum laevigatum* | 0 | 1 |
| Shrub | *Melaleuca squarrosa* | 1 | 0 |
| Shrub | *Monotoca elliptica* | 2 | 1 |
| Shrub | *Olearia rugosa ss. Allenderae* | 2 | 0 |
| Shrub | *Pultenaea daphnoides* | 2 | 0 |
| Shrub | *Spyridium parvifolium* | 9 | 10 |
| Shrub | *Tetratheca ciliata* | 7 | 4 |
| Shurb | *Leptospermum juniperinum* | 2 | 5 |
| Shrub tree | *Bedfordia arborescens* | 0 | 1 |
| Shrub tree | *Hakea decurrens* | 4 | 5 |
| Shrub tree | *Lomatia fraseri* | 0 | 1 |
| Understory tree | *Pomaderris aspera* | 3 | 7 |

*Indicates species that are listed in the Ecological Vegetation Class benchmark for Shrubby Foothill Forests of Wilsons Promontory

*^A^* Growth forms were retrieved from the AusTraits database and the most common form listed for the species was used for this grouping.

FIGURE S5: Alternative sites were dominated by structurally homogeneous mid-story vegetation (20–200 cm), whereas reference sites showed greater consistency in overstory and ground cover (0–20 cm). PC1 (28.9% variance) captured this gradient, separating sites with high mid-story cover from those with stronger canopy and ground-layer structure. PC1 scores differed significantly between states (p = 0.05), consistent with dissimilarity-based tests.


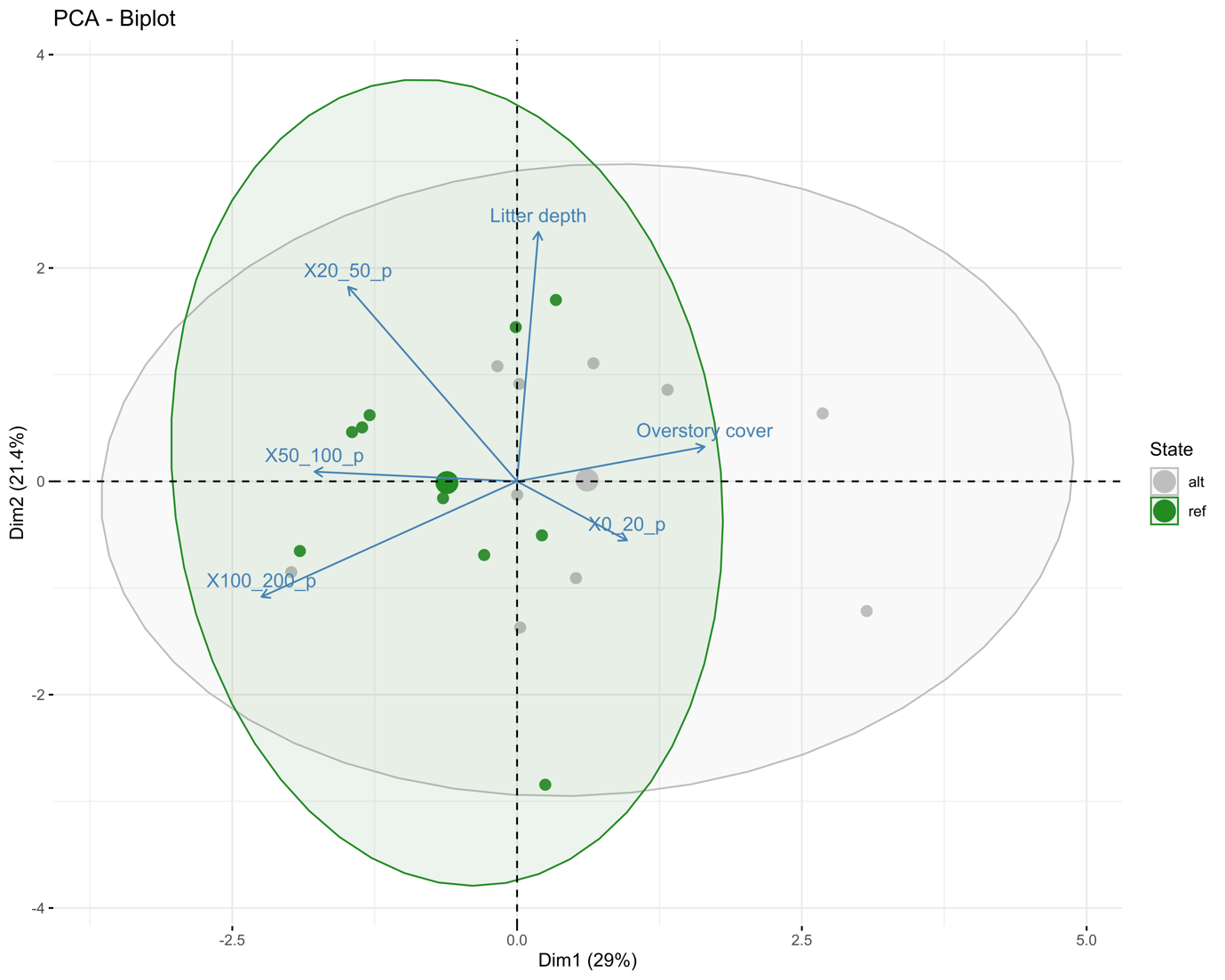

Supplement: Supplementary file 1 — Appendix S1: gcb70895‐sup‐0001‐Supinfo.zip. [file GCB-32-e70895-s001.zip › gcb70895-sup-0001-FigureS1-S5-TableS2-S4@Heap_et_al_supplementary_Oct_2025.docx]
